# Embracing nature’s complexity: Immunoparasitology in the wild

**DOI:** 10.1016/j.smim.2021.101525

**Published:** 2021-03

**Authors:** Iris Mair, Tom N. McNeilly, Yolanda Corripio-Miyar, Ruth Forman, Kathryn J. Else

**Affiliations:** a Lydia Becker Institute of Immunology and Inflammation, Faculty of Biology, Medicine and Health, The University of Manchester, Manchester Academic Health Science Centre, Oxford Road Manchester, M13 9PT, UK; b Disease Control Department, Moredun Research Institute, Midlothian, EH26 0PZ, Scotland, UK

**Keywords:** BTB, bovine tuberculosis, KNP, Kruger National Park, FEC, faecal egg count, PWM, pokeweed mitogen, Immunoparasitology, Type 2 immunity, Ecoimmunology, Field studies, Host-parasite interaction, Population ecology

## Abstract

A wealth of research is dedicated to understanding how resistance against parasites is conferred and how parasite-driven pathology is regulated. This research is in part driven by the hope to better treatments for parasitic diseases of humans and livestock, and in part by immunologists who use parasitic infections as biomedical tools to evoke physiological immune responses. Much of the current mechanistic knowledge has been discovered in laboratory studies using model organisms, especially the laboratory mouse. However, wildlife are also hosts to a range of parasites. Through the study of host-parasite interactions in these non-laboratory systems we can gain a deeper understanding of parasite immunology in a more natural, complex environment. With a focus on helminth parasites, we here explore the insights gained into parasite-induced immune responses through (for immunologists) non-conventional experimental systems, and how current core findings from laboratory studies are reflected in these more natural conditions. The quality of the immune response is undoubtedly a central player in susceptibility versus resistance, as many laboratory studies have shown. Yet, in the wild, parasite infections tend to be chronic diseases. Whilst reading our review, we encourage the reader to consider the following questions which may (only) be answered by studying naturally occurring parasites in the wild: a) what type of immune responses are mounted against parasites in different hosts in the wild, and how do they vary within an individual over time, between individuals of the same species and between species? b) can we use wild or semi-wild study systems to understand the evolutionary drivers for tolerance versus resistance towards a parasite? c) what determines the ability of the host to cope with an infection and is there a link with the type of immune response mounted? d) can we modulate environmental factors to manipulate a wild animal’s immune response to parasitic infections, with translation potential for humans, wildlife, and livestock? and e) in context of this special issue, what lessons for Type 2 immunity can we glean from studying animals in their natural environments? Further, we aim to integrate some of the knowledge gained in semi-wild and wild settings with knowledge gained from traditional laboratory-based research, and to raise awareness for the opportunities (and challenges) that come with integrating a multitude of naturally-occurring variables into immunoparasitological research.

## Highlights

• Ecoimmunology allows the study of parasite immunity in the context of genetic and environmental variation.
• Studies in wild populations highlight the interactions between multiple variables.
• Poor availability of immunological tools has hampered progress in non-model species.
• Wild animal studies combine insights into host immunity and effects at population level.
• We propose a virtuous cycle of testing hypotheses in both lab and wild systems.

## Graphical abstract

**Figure:**
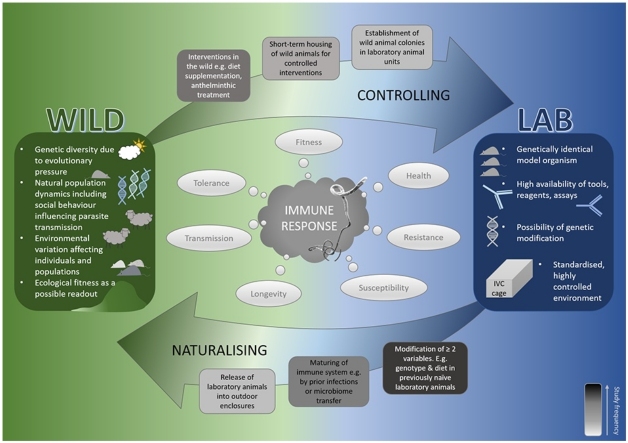

## 1 Introduction

### 1.1 Why do we want to study parasite resistance?

Parasites by definition live in or on other living organisms causing harm to their hosts. Thus, one of the key drivers for studying resistance to parasites is the need to understand, minimise and/or eliminate that harm. For humans, parasites of both plant and domestic animals pose enormous economic burdens within the agricultural industry, driving research which strives to protect the host from infection [1]. Parasites of man cause significant morbidity and mortality globally with few parasitic diseases benefiting from vaccine-mediated resistance. Conservationists also add to the drive to study parasite resistance. The elevation in extinction rates seen in modern species presents a biodiversity crisis [2,3] and whilst the reasons for the growing number of species threatened by extinction are complex and multifactorial, with anthropogenic disturbance a major player, parasitic diseases, as exemplified within amphibians [4,5] are also at play.

Linking the two together, as is particularly evident in for example Africa, the change in land use in order to secure food resources for a growing population, has led to widespread conversion of wildlands for agricultural and pastoral use. Thus, wildlife and livestock are increasingly managed in mixed-communities. Consequently, these more frequent interactions between wildlife and domestic animals have the potential to impact parasite and microbe transmission dynamics. Biodiversity loss and land use change certainly appear to increase infectious disease transmission [6] between wild and domestic animals. The potential for cross-infection between livestock and wildlife has been demonstrated in several studies [7,8], raising the risk of zoonoses emerging between humans and animals. Whilst much basic research ultimately aims to translate findings to the clinic, the veterinary world or in the context of wildlife, a significant volume of work also employs parasites within their hosts as tools to “probe” the immune systems. Thus antigens are delivered in a physiological way to the host enabling an exploration of the subsequent immune responses. The diverse applications of immunoparasitological research and the complex effects of parasitic disease on connected animal populations places this research area well in the concept of ‘One Health’ [9], a holistic research approach that acknowledges the interdependence of one species’ health with the overall health of the ecosystem it lives in.

### 1.2 The early parasitology research – before the laboratory mouse

In order to appreciate how we came to use the study systems we use today for immunoparasitological research, it is worth looking at the history of this research area. Before the establishment of well-defined model organisms, the experimental system was of course humans themselves. Parasitological as well as immunological knowledge originate from careful observation of natural variation in health and disease and the attempt to understand the link between parasite occurrence and health outcome. Descriptions of parasitic infections can be traced back as far as 3000 years B.C. from Egyptian medical papyri [10]. The earliest documentation of experimental interventions come from ancient Arabic societies, where Oriental sores (now known to be caused by the parasitic protozoan *Leishmania* spp.) were widespread. It became common practice to expose the buttocks of especially young girls to exudates from somebody else’s active lesion. Such practice was based on the anecdotal observations that a lesion would only form once in a life, so – without the knowledge of the mechanism or causative agent – the intervention was used to prevent the formation of a potentially disfiguring scar in a more noticeable place such as the face. This procedure was likely one of the first recorded attempts to proactively stimulate an immune response against a parasite [11]. Compared to microscopic infective agents, macroparasites had of course been observed and potentially linked to disease for centuries, if not millennia. However, it was not until the 19th century that the causative agents of disease were discovered with the establishment of the germ theory by Louis Pasteur, the discovery of viruses by Pierre-Paul Emile Roux, Robert Koch’s postulates associating microbes with disease, and Patrick Manson’s ground-breaking work discovering intermediate hosts for parasitic infections. In the 19th and 20th century, several self-experiments or experiments with human volunteers progressed understanding of parasite life cycles and transmission [12]. These early human experiments, while sometimes leading to ground-breaking discoveries or theories, have of course, from today’s viewpoint, little scientific weight due to lack of reproducibility, and would be considered highly unethical. It was only in the 1950s that standardized experimental infection protocols in human volunteers were established, applying modern trial designs with control groups, randomization and much larger sample sizes – and importantly, ethical guidelines [13].

In the meantime, researchers turned to model systems to study the immune response to parasites and how resistance was conferred, with an emphasis on understanding human disease. In the late 19th century, William MacCallum used birds infected with the malaria-related parasite *Haemoproteus columbae* discovering sexual stages in the blood, while Ronald Ross unraveled the whole transmission cycle of *Plasmodium relictum* using culicine mosquitos and infected birds [14]. However, over time, the laboratory mouse became established as the workhorse for immunological research which meant that a growing number of immunoparasitological studies were conducted in laboratory mice [15]. Advances in research showed that different types of parasites require very different types of immune responses to mediate resistance. This is exemplified by the quality of the T helper cell response required to combat intracellular protozoan parasites (T helper 1) versus multicellular extracellular parasites (T helper 2) [[16], [17], [18]]. Whilst contemporary immunology would view the T helper 2/ T helper 1 paradigm as somewhat over-simplified, it still provides a useful framework upon which to understand the immune response to parasites. Indeed, in the context of T helper 2 (or Type 2) mediated immunity there is a long history and breadth of knowledge surrounding resistance to gastro-intestinal dwelling nematode parasites and much of this has been built around the study of immunology in model organisms [[19], [20], [21], [22]]. Given this rich background literature, we have chosen to focus this review primarily on gastro-intestinal dwelling nematodes, and how our understanding of the immune response to parasites has been enriched by studies in non-laboratory systems.

### 1.3 The choice of model systems: why study immunoparasitology in the laboratory mouse?

House mice (*Mus musculus*) are excellent for the study of immunity to gastro-intestinal nematode infections as they provide one of the most complete “immunological toolboxes” available for the dissection of immune responses. The availability of the full genome sequence and inbred and congenic mouse strains add to this power. Transgenic mouse strains exist in a variety of flavors. The term “transgenic mouse” embraces global “knockouts” – where all mouse cells lack a particular immune factor (e.g. a cytokine or a cytokine receptor) and cell specific knockouts, where the immune deficiency is restricted to a particular cell type (for example a macrophage or an epithelial cell). Further, cell-specific deficiencies can be induced. Here a particular deficiency is manipulated to occur only at a particular time in an animal’s life, or at a particular time point post infection [23,24]. Factors such as cytokines can also be overexpressed in transgenic mice, allowing not only a downregulation but also upregulation of specific pathways. Benefitting from the widespread use of the laboratory mouse in pure immunological research, our knowledge of the immune cell types and cellular networks within the immune system is advanced. It is through laboratory mouse studies that we have learnt that modified, or regulated, Type 2-driven effector mechanisms are associated with chronic gut dwelling nematode infections, with T-regulatory cells functioning to ameliorate Type 2 driven inflammatory damage [25]. Studies on immunity to infection in the laboratory mouse have also enabled the clear demonstration that the context within which an infection is experienced matters. Laboratory studies are usually undertaken on juvenile, genetically identical mice, of the same age and sex, kept in homogeneous conditions with an unlimited supply of food. However, changing the sex, the age, the diet, or other variables of the experimental setup, can alter the immune response and outcome of infection [[26], [27], [28]]. This has been well exemplified within laboratory mouse studies, some of which we highlight in Table 1.

**Table 1:** Introducing variation to the laboratory mouse during helminth infections.

Variable	Observation	References
Diet	Protein deprivation enhances susceptibility to *T. muris* and *H. polygyrus* accompanied by a reduced Type 2 immune response, in one study strain-dependent	[88,127,128]
	Zinc deficient mice suffer prolonged infections with gut nematode infections and depressed Type 2 immune responses	[89,129,130]
	A high fat diet leads to increased resistance to *T. muris* infection and reduced Type 1 responses	[90]
	Fermentable dietary fibre prevents expulsion of *T. muris* and heightens the Type 1 response	[91]
	During protein deprivation, co-infection affected parasite survival and fitness via host immunity	[131]
Age	18−28 months old mice are less able to clear gut nematode infections and have reduced Type 2 immune responses compared to 3 month old mice	[28,132]
Sex	Female mice are more resistant to *T. muris* infection associated with elevated Type 2 cytokines	[26]
	BALB/c female mice are more susceptible to *L. sigmodontis* infection	[133]
Genetics	Inbred strains of mice differ in their ability to expel gut nematode infections	[27,134,135,136]
	Resistance to helminth infection is associated with genes within the Major Histocompatibility complex	
Microbiome	Helminths can upregulate microbiota-derived short chain fatty acids correlating with reduced Type 2 immune responses and increase regulation	[137]
	*N. brasiliensis* infection reduced segmented filamentous bacteria, reducing Th17 responses via a Type 2 mechanism	[138]
	During a high fat diet, *H. polygyrus* induces gut microbiota changes via a Type 2 immune response, affecting obesity	[139]
	*H. polygryus* ameliorates viral lung pathology via a microbiome dependent upregulation in Type 1 interferons	[140]
	Multiple helminth species enrich the bacterial species *Lactobacillaceae* with varied outcomes on worm persistence according to the helminth species	[65,66,67,68,69,70,141]
	Genetic and sex differences impact on how the gut microbiota responds to helminth infection	[142]
Infection history	Single high dose infection of between 100−400 *T. muris* eggs results in a dominant Type 2 immune response and worm expulsion in most mouse strains	[135]
	Low dose infection (<50 eggs) results in a Type 1 immune response and susceptibility to *T. muris* infection	[143]
	Repeated low dose (“trickle”) infections enables a build-up of Type 2 immunity and ultimately worm expulsion; though outcome is influenced by genetic background	[44,143]
	Strain-dependent, trickle infections with *H. polygyrus* result in a declining worm burden after several weeks	[144,145]
	Helminth co-infections shapes immune response to future infectious challenges	[146,147,148]

Underlined further variables altered beyond the main variable denoted on the left-hand side.

Laboratory mice kept in controlled, clean conditions have profoundly different immune responses from their wild counterparts [29], which live in complex natural surroundings where coinfections are the norm and the many sources of variation that are removed in the lab, have free play. A benefit of laboratory settings is that they do allow for small numbers of environmental or host-intrinsic variables to be manipulated in a controlled way in the context of specific infections. These controlled model systems have enormous utility, however they cannot, and do not aspire to, fully recreate the effects of multiple variables, life history or the evolutionary pressures that an animal’s immune system will experience in natural settings, i.e. we are unable to appreciate life in its full complexity [30]. As exemplified in several studies mentioned in Table 1, multiple experimental variables can interact with each other. In wild animal populations, the environment is physically undefined with known unknowns and indeed unknown unknowns. Diet, age, infection history, genetics, along with multiple other host-extrinsic and -intrinsic factors, combine to determine the character of an individual’s immune response to an infection. Further, these complex interactions may be cumulative or synergistic in certain settings. For certain questions, it is worth embracing the “messiness” of wild or more natural study systems. We will in this review focus on two groups of animals which are leading the field (for wild studies) in the context of analyses of immune responses to parasitic infections – rodents, with special emphasis on *Mus musculus*, *Apodemus sylvaticus* and *Microtus agrestis,* and ungulates, with a special focus on *Syncerus caffer*, *Equus quagga* and *Ovis aries*.

## 2 Introducing the “wild” study systems: rodents in more natural environments

**Fig. 1:**
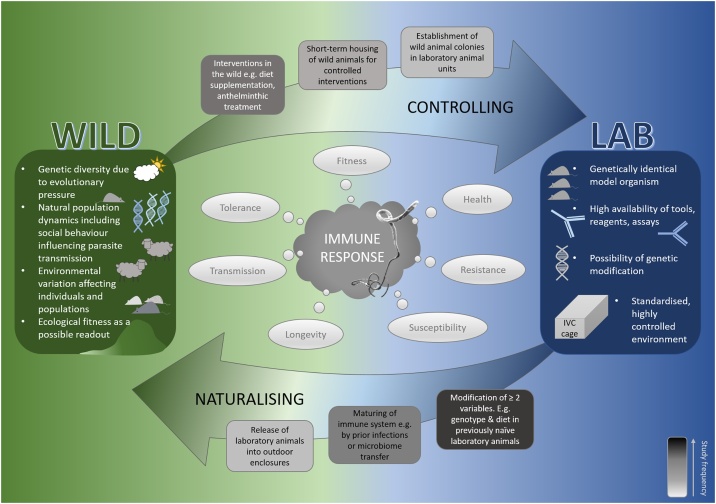
**Bridging the gap between the lab and the wild and back to the lab – generating hypotheses from the wild to explore in the lab and hypotheses in the lab to confirm in the wild.** Experiments have been undertaken for hundreds of years in order to understand the causes and consequences of the type of immune response mounted and/or parasite resistance. Laboratory and field studies have seemingly opposing strength, and may focus on slightly different questions, yet the gap between the two is bridged by study designs that either increase the control in wild study populations or increase environmental variation in laboratory populations.

Over the past decades, several flavours of immunological study systems involving *Mus musculus* have been established aiming to bridge the gap between the lab and the wild. These studies heralded the birth of a new interdisciplinary field formed from ecology and immunology termed “ecoimmunology” (see Fig. 1). At the far end of the spectrum, free-living populations of *Mus musculus* are being studied in their natural environments. In some studies these mice associate closely with human habitation [31], whilst in others they are relatively independent of anthropogenic influences, as is the case on sparsely or uninhabited islands such as Skokholm [32] and the Isle of May [[33], [34], [35]]. Pet shop mice have also served as study system to look at the immune system in a less controlled environment [36,37]. In addition, and rather than starting with mice in the wild, the other end of the ecoimmunology spectrum involves naturalising laboratory mice. Here, laboratory mice are released into outdoor enclosures, allowing for the control of genetics, age and juvenile life-history, but incorporating environmental variables into the system [38]. The introduction of ‘wild factors’ into laboratory mice is another tool that has been used in several ways to mature the laboratory mouse immune system in a more physiological way – from co-housing laboratory mice with pet shop mice [36], transferring of microbiomes from wild mice into germ-free laboratory mice [39], to transplanting laboratory mouse embryos into wild mouse recipients [40]. It is worthwhile mentioning that, while not *Mus musculus*, another elegant comparative study system between the lab and the wild has been developed with wood mice, *Apodemus sylvaticus* [41]. Here, two colonies derived from a third local wild wood mouse population have been established in the lab, one treated conventionally for most infections, the other one re-derived by implanting embryos of wild mice into clean, laboratory surrogate mothers. Investigating immune responses to parasites across those two laboratory colonies and their wild counterpart is yet another approach to disentangle the environmental variables that influence the immune response. The obvious benefit of studying naturalised house mouse models is of course the in-depth knowledge of the immunology and overall physiology of this species, coupled with the availability of experimental tools and assays, and the potential to bridge from the lab to the wild. However, the ecological context (e. g. parasite load and diversity, diet, genetic heterogeneity) of any one study population is critical in enabling specific immunological questions to be asked; certain questions may be better addressed in rodent species other than *Mus musculus*. Further, successful translation of findings from just one species to human health or other wild animals remains rare, and a breadth of study systems is likely to lead to more robust insights into the dynamics of the immune system.

### 2.1 Re-wilding the laboratory mouse and the impact on Type 2 immunity

In the context of extending our knowledge of Type 2 immunity, arguably the most advanced of these new ecoimmunological experimental systems is the re-wilding of laboratory mice [42]. One of the early pioneers of re-wilding laboratory mice was Scott (1991) [43] who explored changes in susceptibility of mice to *H. polygyrus* under more natural settings. Employing genetically susceptible C57BL/6 mice and releasing them into (small) indoor arenas, where transmission rates of the parasite could be manipulated, revealed that C57BL/6 mice became less susceptible to infection at lower transmission rates. Whilst the quality of the immune response was not explored in this study one might infer from, for example, low level “trickle” exposure regimens employed for other helminth species in laboratory mice [44], that a Type 2 immune response had developed.

Developing this re-wilding approach further, Graham built a large outdoor area to home laboratory mice [42]. Consisting of 8 different predator free arenas, natural vegetation and an environment where the mice experience fluctuating temperatures, this system presents a powerful opportunity to explore the effects of the environment on the immune response. Thus, genetic variation can be precisely controlled for in the context of a variable environment. Importantly, it also offers the possibility of longitudinal sampling, which can be challenging in fully wild systems where recapture rates are sometimes low and decay with time. In the context of Type 2 immunity to helminths, one of the most informative studies to date revealed that C57BL/6 mice, which are normally resistant to *T. muris* under standard laboratory conditions, were significantly more susceptible to infection when “re-wilded” into a natural outdoor environment [45]. The change in parasite expulsion phenotype was accompanied by a lowering of the Type 2 immune response and an elevated Type 1 response. Of course the re-directing of the Type 2 immune response towards Type 1 is entirely predictable from laboratory studies given the change in expulsion phenotype in these mice [46], although the environmental driver of this change is as yet unknown. Perhaps more surprisingly, this study showed that not only did mice released into the wild *prior* to receiving *T. muris* eggs become more susceptible to infection than laboratory-maintained C57BL/6 mice infected in the laboratory, but so did C57BL/6 released into the wild at 2 weeks post infection. This latter observation is perhaps more surprising given that the priming of the immune response during the first two weeks of infection occurred within a conventional laboratory environment. Both groups of re-wilded mice had altered gut microbial communities compared to laboratory maintained C57BL/6. Further, and whilst we tend to still be bacteria-centric in studies exploring the impact of the microbiome on multiple parameters of host life, the semi-wild system also led to the discovery that fungi are key players in shaping the immune response [38]. Building on this complexity, the same group went on to show that the changes in the microbiome observed during rewilding was somewhat “buffered “by the presence of *T. muris* infection which stabilized the presence of certain bacterial species [47]. Thus, any controlled creation of “realism” has to capture complexity apparent in both internal and external ecosystems [42,48].

### 2.2 A glimpse into responses to parasites in natural rodent populations – lessons for Type 2 immunity?

#### 2.2.1 Genetics and immunity

The exposure to a wide range of pathogens across a lifetime is likely to result in a complex selection pressure on immune genes within wild animals, rendering them ideal to study immunogenetic selection. Whilst variation in MHC genes has been the subject of extensive research [49], more recently other immune genes such as cytokine genes have been studied. One study found that particularly *Il1b* and *Il2* displayed genetic diversity in a field vole population [50], and further that these polymorphisms were linked with parasite burden, to a similar extent as other host intrinsic factors such sex and body weight [51]. Further, rodents in particular, with their short generation lifespan and the comparatively frequent population crashes, lend themselves to the study of immune gene selection processes longitudinally within a feasible time frame. Of course, this also cautions a direct translation to mammals with differing selection pressures.

An elegant way to address the influence of host genetics on parasite resistance is to use a natural divergence in host genotype as occurs in the house mouse hybrid zone (HMHZ) between Western Europe (*M. musculus domesticus*) and Eastern Europe (*M. musculus musculus*). As the hybrid males are sterile, there is a strong central barrier to gene flow in this zone [52]. The effect of hybridization on resistance has been debated but more recently has been proposed to correlate with less diversity and lower load of intestinal helminths compared to ‘pure’ individuals [53]. A later study interrogated the involvement of the microbiome and the immune system in the differing parasite response between the two subspecies and the hybrids, and found a genetic basis for aberrant microbiome communities, partially explained by differing immune gene expression in the intestine [54]. This study also accounted for sex and reproductive status (pregnancy), neither of which had a significant impact on these measures. From an immunological point of view, some gene expression patterns stood out as being particularly interesting. Highlights included genes whose expression in the gut decreased with increasing interspecific heterozygosity: *Tbx21* and *STAT4*, both required for Th1 differentiation; the immune suppressive/modulatory cytokine *TGFb*; *CCR7 and VCAM1*, important for T cell homing and migration; and *CD28* and *CD3e*, required for T cell activation. The reduction in Type 1 immune gene expression fits with the earlier observed reduction in worm burden in the hybrids [53]. Excitingly, this study analysed laboratory-kept inbred *domesticus* and *musculus* strains and their hybrids in parallel, and several of the QTLs and immune gene expression patterns identified in the wild study were also found in the laboratory study. These types of complementary studies allow for the cross-fertilization of ideas and strengthening of findings between the wild and the lab.

#### 2.2.2 The influence of coinfections on immune responses

In naturally occurring populations, including humans, coinfections rather than isolated infections are the norm. Studying the relationships of parasites with each other and the host immune system, within a natural host, gives a starting point for understanding the broad dynamics at play, within a varied context. Utilising a wood mouse population naturally infected with both *H. polygyrus* and the coccidia *Eimeria* spp., a study established a positive relationship between *H. polygyrus* specific serum IgG1 levels and parasite burden, however, the presence of *Eimeria* had a negative influence on helminth-specific antibody levels [55]. A follow-up interventional experiment using a laboratory-kept wild wood mouse colony noted that during coinfection with *H. polygyrus* and *Eimeria* spp., *H polygyrus* dampened *Eimeria* fitness as assessed by oocyst shedding whilst simultaneously benefitting from the coinfection, as the helminth exhibited higher egg shedding and delayed expulsion compared to control animals infected with just *H. polygyrus* [56]. Understanding the mechanistic basis for this parasite interaction is likely to require more laboratory-based interventional approaches, however it is tempting to speculate that a modulated T helper cell response is involved.

#### 2.2.3 Studying the immune system in wild mice

Without a particular emphasis on the infectious agents involved, the detailed study of immune cells in feral house mice has contributed enormously to our appreciation that the immune system of naïve laboratory mice is indeed naïve, in that it is more akin to the immune system of a newborn than that of an adult human [36]. The difference in phenotype and abundance of certain immune cells or cell subsets in wild house mice has been shown for T cells [31,36], B cells, myeloid cells [31] including eosinophils [35], and NK cells [31,57]. Antigenic exposure is deemed one of the main drivers of these changes, although the immune variation observed in wild mice is likely the result of a more complex network of influencing factors. Immune function has been probed in several wild rodent studies using *ex vivo* stimulations [31,57,58], immunisation with sheep red blood cells [[59], [60], [61]] or keyhole limpet haemocyanin [62], or via bacterial challenge [36]. Interestingly, cytokine production in both innate immune cells as well as T cells has been reported as altered in feral compared to laboratory rodents, suggesting a functionally different state of the immune system beyond their altered phenotype.

Beyond the analysis of CD8^+^ T cells in cervical tissue [36] and of eosinophils in peritoneal exudate [35] of wild mice, the non-lymphoid tissue distribution and phenotype of immune cells in wild mice living with multiple infections is as yet unexplored. The classical yet debated classification of Type 1 and Type 2 responses with their respective markers would be an ideal candidate to explore in house mice living under natural conditions.

#### 2.2.4 Exploring the microbiome

Building on the observations in semi-wild systems which reveal a complex interplay between host, parasite and microbiome, fully wild systems are making their own unique contributions. For example, in wild *Apodemus flavicollis*, the presence of helminth parasites contributed to microbiota diversity, with each individual helminth having a different effect on the microbiome composition [63]. Effects were seen both downstream and upstream of the helminth’s niche. Specifically, *Syphacia* abundance was associated with a decrease in Lactobacillus OTUs, a type of bacteria with immunomodulatory capacities [64]. Notably, no other helminths had significant associations with these bacteria, whereas multiple types of helminth infections have been reported to enrich bacterial species within the family Lactobacillaceae in laboratory studies [[65], [66], [67], [68], [69], [70]]. With an increasing interest in the factors that shape wild rodent microbiome composition and its effect on host fitness [34,[71], [72], [73], [74], [75], [76]], these wild studies may help add to the diverse and sometimes contradicting laboratory findings [77].

#### 2.2.5 Teasing apart diet, anthelminthic treatment and immune interactions

While several wild rodent studies utilize anthelminthic treatment in order to understand the consequences of (co-)infection for host fitness, the interaction amongst parasites, or within-population transmission [[78], [79], [80], [81], [82]], the immune response has rarely been described in these studies. This is a clear opportunity for further immunological research, as anthelminthic treatment is the main and often only intervention for people living in parasite endemic areas. Understanding which factors modulate the immune response to the parasite, treatment efficacy, and drug-immune interactions, would be highly desirable. A study delivering anthelminthics to wild wood mice showed treatment was more efficient in adults compared to juvenile mice [56]. Importantly, mouse age affected the directionality with which helminth burdens at first capture predicted subsequent parasite-specific IgG1 levels, demonstrating an interaction between age, parasite burden and immunity. An experimental set-up more akin to laboratory studies was performed using *ex vivo* stimulation of mesenteric lymph node cells (local response) and splenocytes (systemic response) of wood mice treated with anthelminthics or left untreated [83]. Splenic TNF-α production was most affected by anthelminthic treatment of the host, being increased in males, but interestingly decreased in females compared to untreated controls. There was also an age-dependent decline in TNF-α production in anthelminthic-treated individuals which was not observed in controls.

Anthelminthic treatment has also been coupled with food supplementation to study the interaction between host condition, infection, and the immune response. Food supplementation of wild wood mice has been shown to lead to reduced *H. polygyrus* worm burdens, egg shedding, and increased anthelminthic efficacy [41]. Whilst there was no direct association with parasite-specific serum IgG1, body condition positively correlated with this immune measure. The authors suggest that diet may influence the immune response via supporting overall body condition. Parallel laboratory studies, using an established wild wood mouse colony, replicated some but not all relationships detected in the wild, allowing for a discussion of the observed phenotypes. Further, wild field voles captured and placed in outdoor enclosures during boreal winter, and subsequently treated with anthelminthics, have also been studied in the context of diet supplementation [84]. Here, total IgG levels and neutrophil-to-lymphocyte ratios were higher in food-supplemented compared to non-supplemented animals, and helminth faecal egg counts were reduced [85]. Again, the quality of the immune response was not dissected beyond analyzing IgG1 levels, but the experimental set up would suit follow-up questions. The overall effect of food supplementation on wildlife immunity is still not well understood [86], but body condition is thought to be a major player in determining susceptibility to infection [87]. Dietary variation as captured by carbon and nitrogen stable isotope ratios was able to explain most of the variation observed in immune gene expression in the mesenteric lymph nodes of wild house mice, beyond age and infection status [33]. Whether diet would also alter parasite-specific responses in the draining lymph nodes, quantitatively or qualitatively, is as yet unknown. As also addressed within controlled laboratory studies [[88], [89], [90], [91]], which aspect of food provisioning is important for the modulation of immune response – caloric supplementation or specific macro– or micronutrients – would be an intriguing future avenue for wild studies.

#### 2.2.6 Seasonal changes in immune responses

The change of seasons, with its manifold associated changes including temperature, risk of predation, food sources, and the inherent impact this has on host social behavior and reproductive state, make it an ideal candidate to study in the wild. Immune investment has been shown to follow a seasonal pattern in field voles, with low investment in the adaptive immune response during winter, as measured by longitudinal lymphocyte counts in the blood. Lymphocyte counts were also adversely affected by body condition and population density [92]. Comparative data collection in a laboratory-kept colony confirmed that these changes were indeed dependent on the seasonal changes of the environment. A later study using the same field vole population employed qPCR on TLR- or mitogen-stimulated splenocytes sampled across seasons [93]. Notably, an analysis of pro-inflammatory gene expression level revealed that pro-inflammatory responses characterised by for example T-bet, IFN-γ and IL-2 were highest in late winter and declined through summer and autumn before increasing again towards winter. This study also highlighted a negative association between the expression of the Type 2 transcription factor GATA3 with the Type 1 transcription factor T-bet as well as IFN-γ and IL-2. These data therefore strongly suggest that a negative feedback loop between these two immune states exists in the wild as it would be inferred to be based on laboratory studies.

The seasonal variation of immune investment in wildlife has been proposed to have evolved to reduce the energetic cost of maintaining immune responses during times in which food sources are not readily available [94,95]. How they are induced and what impact it has on the quality of the immune response to infections for an individual in its specific context, remains to be explored.

#### 2.2.7 Tracking immune responses over time in the wild

Arguably one of the most directed analyses of the link between the immune response and parasitic infections in the context of tolerance or resistance over time was performed using a population of field voles in the Kielder forest [96]. This study sampled almost 1000 animals longitudinally and 600 cross-sectionally. Tolerance in the ecological sense is any mechanism that allows an individual to mitigate, i.e. tolerate, high infection levels. These can be immunological in nature, but do not have to be. Thus immunological tolerance is one of the possible means of inducing (ecological) tolerance to infection, but equally, the expression of serpins, for example, that target bacterial proteases, or other host responses that dampen pathogen virulence would fall under that category [97]. The authors discovered that young males were prone towards resistance, with decreasing parasite burdens over time, whereas mature males were more likely to display a tolerance pattern, with a slow accumulation of parasites [96]. Tolerance in older individuals correlated with better overall condition, judged by a modified body mass index. Importantly, expression of GATA3 – a transcription factor associated with Type 2 immunity – in mitogen-stimulated splenocytes correlated positively with body condition in mature, tolerant males, but not young males with a resistance phenotype. Also, GATA3 expression could predict age-specific survival rate but simultaneously negatively correlated with a proxy for reproductive fitness. These patterns suggest that in this population, parasitic infection may lead to a trade-off between host health and investment into reproduction, both energetically costly processes. Turning to the longitudinal data, macroparasite infection appeared to precede GATA3 expression which in turn preceded weight gain, suggesting that a causative relationship may be at play between a Type 2 response and investment in body condition. Due to the observational nature of most wild studies, it is of course only an indication, but longitudinal sampling does allow the investigation of temporal patterns of variables of interest. The proposition of enhanced GATA3 expression as a biomarker for tolerance, rather than of imminent parasite expulsion as would be assumed in a laboratory setting, is something to ponder about. In humans infected with certain gastro-intestinal helminth species, children are more susceptible than adults, and in chronically infected adults, often a slow increase in Type 2 immunity and reduced parasite load can be observed. One could argue that from an evolutionary perspective, the shorter lifespan of rodents means that it is not worth investing in resistance mechanisms against macroparasites once adulthood is reached, as the high reproductive rate during warmer seasons and food scarcity during winter may limit the resources for immune investment. However, this simplified model of tolerance versus resistance strategies is debated [97].

## 3 Introducing wild ungulate study systems

Wild ungulates are constantly exposed to parasite challenge and have distinct advantages for the study of immunity to parasites. Not only are they exposed to multiple pathogens which may affect or be affected by parasite immune responses, they exist in environments where seasonal changes take place affecting parasite exposure. Furthermore, these studies significantly benefit from cross-reactive immunological reagents from the veterinary immunology toolbox to allow measurement of immune phenotypes including T cell responses and immune effectors of the Type 2 response. A major benefit of these study systems is that long-lived ungulates can be recaptured over multiple sampling time-points, allowing the repeatability of both parasitological and immune measurements to be investigated. Such individual based monitoring is also possible over the life-time of the individual, allowing the influence of parasite immunity on survival and life-time reproductive success (evolutionary fitness). Furthermore, the larger size of wild ungulates allows for larger sample volumes to be collected, which could be challenging when working with wild rodents. A number of different systems have been studied with distinct advantages and disadvantages as described below.

### 3.1 African buffalo system – a wild study population with defined co-infecting pathogens

A population of wild African buffalo (*Syncerus caffer*) in the Kruger National Park (KNP) in northeastern South Africa has been used in a number of studies exploring various aspects of Type 2 immunity. Importantly bovine tuberculosis (BTB) is endemic in this population, and as BTB is largely controlled by Type 1 immune responses [98,99], many of the studies in these Buffalo have specifically focused on the interplay between Type 1 and Type 2 immune responses. Out of a total population of approximately 30,000 free-ranging animals, a few hundred individuals can be captured and recaptured following chemical immobilization through darting, allowing blood and faecal sampling for immune response and parasitological measurements, respectively, and also the potential to administer anthelminthic drugs to clear gastro-intestinal parasites from certain individuals. Importantly, immune reagents developed for use in domestic cattle are cross-reactive in African buffalo due to high degree of homology (e.g. > 95 % for IL-4 and interferon-γ at the amino acid level), allowing measurement of key cytokines and other immune effectors in blood and tissues.

Key findings from this study population have largely focused on understanding the interplay between intestinal nematode infections and BTB. For example, a study of 209 initially BTB negative adult female buffalo tracked over a four-year period demonstrated that animals with greater natural resistance to worms based on strongyle parasite faecal egg counts (FEC) were more likely to die from BTB than non-resistant individuals, and that BTB disease progression was more rapid in worm resistant individuals [100]. The authors demonstrated in a subset of animals that the worm resistant phenotype was associated with increased mucosal mast cells, eosinophils and IgA in the abomasum (gastric stomach) and small intestine, the primary sites of infection by strongyle parasites, which is consistent with a more pronounced Type 2 immune response in resistant individuals. The authors speculated that these enhanced Type 2 responses in worm resistant individuals may have resulted in concomitant down-regulation of Type 1 immune responses which are known to be important for control of BTB, explaining the increased BTB susceptibility in worm resistant animals. These results are consistent with similar studies in humans linking worm infections to TB severity [101,102].

A similar study on the KNP buffalo population evaluated the impact of worm clearance via a long acting anthelminthic bolus on BTB infection risk, mortality and persistence [103]. Worm clearance resulted in enhanced Th1 immune responses, as determined by the magnitude of IFN-ɤ release from whole blood following stimulation with pokeweed mitogen (PWM), and reduced mortality after BTM infection, again suggesting that worm induced Type 2 immunity down-regulated the Type 1 responses. However, rather counterintuitively, the study found increased transmission of BTB in anthelminthic treated buffalo, reflected in an approximately eightfold increase in the basic reproduction number (*R*_0_). As worm clearance did not affect the probability of individuals acquiring BTB, the explanation for these results was that anthelminthic treatment allowed BTB infected individuals to live longer and shed the bacteria over more prolonged periods, resulting in enhanced transmission of the pathogen.

This population has also been used to investigate some assumptions regarding Type 2 immunity and resistance to helminths. In a study of acquisition and clearance of schistosomes in KNP Buffalo, it was found that clearance of schistosomes was associated with lower Type 2 responses as determined by release of IL-4 from PWM stimulated whole blood [104]. Given that immunity to schistosomiasis in man is associated with a Type 2 immune profile including eosinophils and IgE [105,106], these results were rather surprising and suggested that only certain elements of the Th2 response are important for schistosome immunity.

A strength of the buffalo and zebra systems is that pathogens for which resistance is associated with Type1 or Type 2 immunity (BTB and worms, respectively) are endemic within the population, allowing questions around Type 1/Type 2 counter-regulation and trade-offs to be addressed. However, this also has the consequence of contextualizing all immunological phenotypes in terms of Type 1 or Type 2 immunity, potentially missing the continuum of response within a population and the influence of other aspects of the immune response (e.g. Treg, Th17 responses).

### 3.2 The Namibian Zebra study system – a population with strong seasonal pathogen dynamics

Similar to the KNP buffalo system, a natural population of plains zebra (*Equus quagga*) in Etosha National Park, Namibia, has been used to study immunity to helminth parasites in the context of an endemic bacterial disease, anthrax, caused by *Bacillus anthracis* [107,108]. Importantly, infection dynamics of both helminths and *B. anthracis* exhibit strong seasonal patterns, with increased gastro-intestinal nematode infection intensity and anthrax outbreaks in the wet season compared to the dry season [109,110]. By capturing and re-capturing individual zebra over sequential seasons it was found that Type 2 immunity exhibited a strong seasonal dynamic, being enhanced in the wet season. Of more interest was that while blood eosinophil counts were negatively correlated with gastro-intestinal nematode egg count as expected, anti-anthrax antibody titers, which were used as a proxy of immunity to anthrax, were negatively correlated with eosinophil count in the wet season (i.e. anthrax season) but not the dry season [107]. The authors suggested that in addition to seasonal variability in *B. anthracis* exposure, increased cases of anthrax in the wet season may also result from Type 2 skewing of the immune response by co-infection with gastro-intestinal nematodes, resulting in less effective immune control of anthrax. This would also be an example of one pathogen driving seasonality of another pathogen.

### 3.3 The Soay sheep – a long-term study population of defined unmanaged wild ungulates

Soay sheep have lived unmanaged for several thousand years in the St Kilda archipelago, 65 km west of the Outer Hebrides, Scotland. Since 1985, sheep in the Village Bay area of Hirta have been the subject of an intensive individual-based study in which sheep are captured and tagged at birth, and on an annual basis in late summer, in order to collect data on variables including weight and morphometrics, and faecal and blood samples for parasitological and immunological analyses, respectively [111]. The population size is also monitored and reveals a characteristic dynamic of periods of slowly increasing sheep numbers followed by over-winter ‘crashes’ in which >50 % of the population can die. Importantly, unlike buffalo and zebra, sheep can be captured without chemical immobilization, facilitating repeated capture of individuals throughout their lifetime. Also, by using genetic and field monitoring, lifetime reproductive success is known and accurate pedigree information is recorded, setting the study system apart from other study systems such as wild mice. This allows the study of parasite immunity in the context of more evolutionary concepts such as reproductive fitness, rather than simply health and survival. Other important distinctions from the previously described African ungulate systems is that the population does not appear to be affected by a major endemic Type 1 pathogen, with the main pathogen pressure exerted by coccidian and nematode parasites [112,113], and in general, due to the long-term nature of the study, interventions such as anthelminthic treatments are not usually considered. However, this means that studies are perhaps less restricted to the Type 1/Type 2 paradigm.

Initial studies of parasite immunity focused on anti-parasite antibodies, and usefully it was shown that measuring antibody responses to the L3 stage of the gastric parasitic strongyle nematode, *Teladorsagia circumcincta*, reflected a general anti-nematode parasite response [114]. In these initial studies it was shown that anti-nematode IgG responses were strongly and consistently positively associated with overwinter survival, suggesting that there are important fitness consequences of variation in strongyle-specific immunity [115,116]. These initial studies on parasite-specific antibody responses, which focused on analysis of samples collected in late summer prior to high-mortality (‘crash’) winters were extended to a 25-year period from 1990 to 2015, which provided further insights into the anti-parasite antibody response. Firstly, age-dependent effects on the relationship were identified between different antibody isotypes, strongyle FEC and survival, with elevated levels of parasite-specific IgA predicting reduced parasite egg counts but no effects on survival in lambs, whereas in adults it was increased parasite specific IgG which was associated with lower FEC, while also positively predicting overwinter survival [117]. Secondly, by looking at longitudinally sampled adult Soay sheep across this time-period (2215 samples collected from 797 individuals) it was shown that within-individual decline in nematode-specific IgG was predictive of mortality independent of FEC and bodyweight, clearly demonstrating immunosenescence occurring in natural populations [114]. Finally, using this extended dataset it was shown that plasma levels of parasite-specific IgG in neonatal lambs (<10 days old) positively predicted first year survival, independent of total IgG [118]. As plasma IgG in lambs of this age of lamb would be exclusively derived from colostrum [119], this provided evidence for an important role for maternally derived anti-parasite antibodies in offspring survival.

The above studies largely focused on nematode-specific antibody levels in plasma which, while shown to be important, is rather an imprecise measure of the parasite immune response. More recently, cellular immune response measurements have been performed on the Soay population to reflect a broader range of immune response phenotypes, quantifying cytokine release from PWM stimulated blood and determining circulating numbers of Th1 (CD4+Tbet+), Th2 (CD4+GATA3+), Th17 (CD4+RORγt+) and Treg (CD4+Foxp3+) polarized T cells [120], which were then used to look for associations with strongyle and coccidian egg counts, macro- and micro-parasites controlled by Th1 and Th2 responses, respectively [121,122]. As predicted from laboratory and controlled experimental challenge studies, it was found that IL-4 and GATA3 negatively predicted gastro-intestinal strongyle nematode FEC, while production of IFN-γ negatively predicted coccidian faecal oocyst count. However, it was also shown that positive rather than negative associations existed between Th1 and Th2-associated immune measures, indicating that Th1/Th2 trade-offs observed in the laboratory may be less readily apparent in more complex natural systems.

This study population has also been used to explore aspects of tolerance rather than resistance to parasites [123]. As individuals are repeatedly sampled, it was possible to provide tolerance estimates for individuals based on how animal weight changed with increasing parasite burden. This study demonstrated that tolerance to gastro-intestinal strongyle parasites varied between individuals, with some losing weight rapidly with increasing infections (low tolerance) and others lost weigh more slowly (high tolerance). Interestingly, animals with higher tolerance had higher lifetime breeding success. The exact mechanisms by which tolerance acts in this population was not determined, but did not appear to be genetically controlled.

### 3.4 What do wild ungulate systems tell us about parasite immunity and what are their limitations?

These studies described above have provided powerful insights into anti-parasite immunity, which may not be possible in rodent systems, some of which have wider consequences for human and farmed livestock populations. For example, studies in buffalo have identified potential unintended consequences of either worm clearance through mass anthelminthic treatments, which may increase transmission of co-infecting pathogens, or selective breeding of livestock for worm resistance, which may increase susceptibility to other pathogens. Furthermore, attempted breeding of livestock for tolerance to worms may be futile if driven by environmental rather than genetic factors. The long-term nature of the Soay sheep system has also uncovered important age-dependent effects of the anti-parasite immune response, and a role for senescence in anti-helminth immunity in the ecology and evolution of natural populations.

A strength of the buffalo and zebra systems is that pathogens for which resistance is associated with Type 1 or Type 2 immunity are endemic within the population, allowing questions around Type 1/Type 2 counter-regulation and trade-offs to be addressed. However, this also has the consequence of contextualizing all immunological phenotypes in terms of Th1 or Th2 immunity, potentially missing the continuum of response within a population and the influence of other aspects of the immune response. The Soay system, which does not have a prevalent Th1 pathogen other than coccidia, has broadened the range of immune phenotypes to capture wider aspects of parasite immunity [120]. However, these wild systems are still compromised with a lack of precision in immune measurements, partly due to gaps within the veterinary immunology toolbox, the lack of known relevant antigen targets required to develop antigen-specific immune assays, and also due to the lack of ability to study immune responses at the site of parasite infection, instead relying heavily on immune measurements from blood as proxies for the mucosal immune response. Another limitation, particularly in the Soay sheep system, is the difficulty of conducting properly-controlled experimental interventions due to either logistical issues, or the need to avoid altering the natural system under investigation. This means that it can be difficult to disentangle cause and effect relationships between environmental, parasitological and immune variables in the study systems.

## 4 Our look to the future

Better understanding of the immune response to infection in wildlife, zoo animals or livestock has direct implications for conservation, ecosystem stability, animal welfare and economic concerns. However, these systems, which for traditional immunologists would be considered ‘unconventional’, offer unique platforms to study the immune system in a natural context, with applicability to human health, with humans representing another ‘wild’ living animal, subject to intrinsic and extrinsic variation [124].

For a long time, these systems have mainly been exploited by ecologists, epidemiologists, geneticists and parasitologists. Over the past decades, however, the interdisciplinary research area of ecoimmunology is building momentum, aiming to tackle how immune variation is shaped throughout an individual’s lifetime as well as on an evolutionary scale [125,126].

Immune responses to known infections or other immune challenges lend themselves as tools to probe an individual’s immune system, and wild immunoparasitology is therefore one of the strongest areas of research within the ecoimmunology community. We believe that it will be of benefit to both communities and the science that results if immunologists and ecologists are able to share resources and scientific approaches. Whilst ecologists seek to explore the uncontrolled variation seen within a population with the aim to uncover broad patterns and associations, laboratory immunology has focused on minimizing both genetic and environmental variation to investigate the cellular and molecular mechanisms at play within an individual by varying only limited factors. Combining the seemingly opposing strengths of these two approaches will help generate a more robust and holistic understanding of how the immune system functions in, and interacts with, the natural world.

At the start of this review we set out five questions for the reader to consider. Whilst we do not as yet have complete answers to these types of questions, we hope this review has demonstrated how they can (only) be addressed in more wild settings.

For example, in the context of this special issue, what have we learnt about Type 2 immunity from studying animals in the wild? Semi-wild systems have provided clear evidence that re-wilding laboratory mice increases the susceptibility to gastro-intestinal nematodes correlating with a decreased Th2 immune responses. In keeping with this, and exploring the mouse hybrid zone, a reduction in Th1 immune gene expression with increasing interspecific heterozygosity associated with a reduced worm burden in the hybrids. However, the Th2 transcription factor GATA3 correlated positively with a tolerance phenotype in adult field voles. Evidence from further field vole studies suggests an increase in Th1 correlates (T-bet, IFN-γ, IL-2) in late winter with a negative association with GATA-3. Further studies in African buffalo support an association between worm resistance and Th2 immunity. In contrast however, using parasite-specific IgG1 as a surrogate of Th2 immunity, food supplementation of wood mice showed a reduction in H. polygyrus worm burden but no association with IgG1.

Of course, studying animals in the wild brings unique challenges not faced in traditional laboratory studies. For example, the requirement for large sample sizes due to inherently noisy data, lack of reagents in non-model organisms, logistical challenges of processing samples at the field site, the frequent occurrence and statistical challenges of missing values, uncertainty of trapping success, potential crashes in populations or difficulty accessing fields sites due to weather or political problems are all factors which are not faced within a laboratory setting. However, with technical advances, as well as advances in bioinformatics, statistical analyses and computational power, the increasing complexity of ecoimmunological datasets has the potential to provide holistic insights into how the immune system is shaped in the natural world. Ecoimmunological studies are able to make an important contribution to assessing the relative contribution of certain factors to explain the variation observed in immunological readouts. It comes without saying that this emerging field thrives on interdisciplinary research, including ecologists, immunologists, parasitologists, microbiologists, geneticists, bioinformaticians, data scientists, and not to forget, fieldwork assistants and volunteers, and many more. We propose a fluid working model whereby questions and hypotheses arising in necessarily more observational studies using natural systems inspire targeted interventional laboratory experiments, and mechanistic or detailed phenotypic insights from controlled laboratory studies are tested for robustness and reproducibility in more “messy” systems (Fig. 1). Large-scale comparative studies across species and across lab-to-wild systems would allow paradigms to be established with more certainty compared to singular studies with potentially conflicting results.

We would also like to take the opportunity to highlight the technical and logistical challenges involved in the sampling and study design using wild animal systems, whereby sample acquisition for one particular question will often take years in order to achieve the statistical power to draw confident conclusions. Furthermore, the power of the study may not come from a mechanistic insight (expected in most traditional immunological studies) but the ‘real-life snapshot’ of patterns and associations that may or may not have been predicted from controlled laboratory studies. We would like, with this review, to share and celebrate the diversity and ingenuity of past and current wild or semi-wild study systems that embrace the “messiness” in order to investigate immune functions in real world settings.

The lesson from wild immunoparasitology for Type 2 immunity? Context matters; let’s strive to provide it and play with it by building bridges across disciplines and study systems.

## Author contribution

IM, KJE, TNM, and YC-M conceptualised and wrote the manuscript. IM and RF conceptualised and designed the summarising figure. All authors revised and gave final approval to the manuscript.

## Funding

This work was supported by a 10.13039/501100000268Biotechnology and Biological Sciences Research Council grant (BB/P018157/1), a 10.13039/501100000265Medical Research Council grant (MR/N022661/1), both awarded to KJE, and a 10.13039/501100000270Natural Environment Research Council Large grant (NE/R01664X/1), awarded to TNM. TNM receives funding from the Scottish Government Rural Affairs, Food and the Environment (RAFE) Strategic Research Portfolio 2016-2022.

## Declaration of Competing Interest

The authors report no declarations of interest.
